# Factors associated with doctors’ knowledge on antibiotic use in China

**DOI:** 10.1038/srep23429

**Published:** 2016-03-24

**Authors:** Yu Bai, Sijie Wang, Xiaoxv Yin, Jigeng Bai, Yanhong Gong, Zuxun Lu

**Affiliations:** 1 Department of Social Medicine and Health Management, School of Public Health, Tongji Medical College, Huazhong University of Science and Technology, Wuhan, 430030, Hubei, P. R. China; 2 Shanxi Women and Children Health Care Center, Taiyuan, 518001, Shanxi, P. R. China

## Abstract

Misuse of antibiotics by the medical profession is a global concern. Examining doctors’ knowledge about antimicrobials will be important in developing strategies to improve antibiotic use. The aim of the study was to survey Chinese doctors’ knowledge on antibiotics and reveal the factors associated with their level of knowledge. A cross-sectional survey was conducted in Shanxi in central China. A total of 761 physicians were surveyed using a structured self-administered questionnaire. A generalized linear regression model was used to identify the factors associated with doctors’ knowledge on antibiotic. Based on a full score of 10, the average score for doctors’ knowledge on antibiotics was 6.29 (SD = 1.79). Generalized linear regression analysis indicated that doctors who either worked in the internal medicine department, who were chief doctors or who received continuing education on antibiotic, had better knowledge of antibiotics. Compared with doctors working in tertiary hospitals, doctors working in secondary hospitals or primary healthcare facilities had poorer knowledge about antibiotics. Chinese doctors have suboptimal knowledge about antimicrobials. Ongoing education is effective to enhance doctors’ knowledge, but the effect remains to be further improved. More targeted interventions and education programs should improve knowledge about antimicrobials, especially for doctors working in primary healthcare institutions.

Resistance to antibiotics is a growing worldwide public health problem^1^,^2^,^3^,^4^, leading to a delay in the administration of effective therapy and increased hospital costs, morbidity and mortality^5^ The widespread inappropriate use of antibiotics is considered as one of the important significant causes of the development of microbial antibiotic resistance^6^,^7^,^8^,^9^. These facts have prompted many to call for improvements in the way doctors’ prescribe antimicrobials to patients^10^,^11^. The extent of the doctors’ knowledge on antibiotic use has been identified as a key factor that affects individual prescribing behavior^12^. Therefore, it is important to better understand the breadth of knowledge doctors have regarding antibiotic use.

Previous surveys have been conducted to assess doctors’ knowledge about antimicrobials in many countries^13^,^14^,^15^,^16^,^17^,^18^,^19^. As China is one such country with a history of serious abuse of antibiotic use^20^,^21^,^22^, and there has been very little research on doctors’ knowledge concerning antibiotic use in China, further research is urgently needed. Previous studies have mainly focused on descriptive analyses of doctors’ knowledge and have failed to identify the key factors that can be used to develop targeted and effective interventions^13^,^14^,^15^,^19^. The present study aims to measure doctors’ knowledge on antibiotic use in China and reveal these factors.

## Result

Table 1 presents study participant characteristics. A total of 761 participants comprising 473 female doctors (62.16%) and 288 male doctors (37.84%) were surveyed in this study. The mean age of participants was 35.55 years, with standard deviation (SD) of 8.49. Of the participants, more than half had achieved a bachelor degree or above, and their mean working duration in hospital was 10.75 years (SD = 9.45). Approximately 90% had received continuing education on antibiotics use. Based on a full score of 10, the average antibiotic knowledge score was 6.29 (SD = 1.79).

Table 2 presents factors associated with physician’s knowledge on antibiotics. There was no significant difference in the mean score according to age (*P* = 0.24), gender (*P* = 0.36), educational level (*P* = 0.38 for Bachelor degree and *P* = 0.58 for Master degree or higher), and working duration in hospitals (*P* = 0.55). Physicians who worked in the internal medicine department had higher mean knowledge scores than that of those who worked in the pediatrics department (*P* < 0.02), in the stomatology department (*P* < 0.01), and in the general practice department (*P* < 0.01). Chief doctors had a significantly higher mean knowledge score compared with doctors with junior title (*P* < 0.01). Doctors who worked in primary healthcare facilities (*P* < 0.01), as well as those who working in secondary hospitals (*P* < 0.01), had lower average scores than those that worked in tertiary hospital. Doctors receiving continuing education on antibiotics had a higher mean score than doctors who did not (*P* < 0.01).

## Discussion

This study is the first in China to more comprehensively assess doctors’ knowledge on antibiotic use. Based on analysis of the doctors’ scores from the questionnaire survey, overall knowledge of antimicrobials was much lower than expected. As a benchmark, the mean antibiotic knowledge score in this study was similar to that reported by a previous study conducted in the Congo^15^, but was lower than the result from a comparable survey conducted in Peru^14^. The results from different countries may differ owing to the doctors’ level of education and the standard of care in the different tiered hospitals. For example in Congo and Peru, the study participants were doctors who worked in tertiary hospitals or in teaching hospitals^14^,^15^. In contrast, participants recruited in the present study were from high technical tertiary hospitals as well as relative low technical level hospitals.

Multivariable generalized linear regression modeling indicated that doctors who worked in tertiary hospitals had a significantly higher mean knowledge score compared with those who worked in primary healthcare facilities and secondary hospitals. This may be attributed to the fact that doctors in tertiary hospitals have many more opportunities for participation in research and national conferences. Nevertheless, the doctors’ knowledge on antibiotic use even in tertiary hospitals is still insufficient. In addition, the knowledge score on antibiotic use of doctors working in primary healthcare facilities was the lowest. As the primary healthcare facilities (community health centers and township hospitals) in China cover a large number of patients, we propose that doctors working in primary healthcare facilities should be selected as the key target group for training intervention.

Other studies have demonstrated that continuing education is an effective intervention to increase doctors’ knowledge on antibiotic use and to improve their prescribing behavior^23^,^24^,^25^. Our study showed that doctors who received training on antibiotic use in the past year had better antibiotic knowledge, consistent with the previous studies. It should be noted however, that even though more than ninety percent of doctors received training in the present study, they still had a suboptimal knowledge about antimicrobials. This indicates that more regular and improved training methods should be actively promoted. Therefore, it will be necessary to review the key factors that influence doctors’ learning on the use of antibiotics so that more effective educational programs can be developed and implemented.

The present study also demonstrated that doctors who worked in internal medicine department had a relatively better knowledge on antibiotics use, which may simply be due to the fact that they prescribe antibiotics more frequently and pay more careful attention to knowledge about antibiotic use^13^. Previous studies have indicated that consulting with senior physicians is an important knowledge source for junior physicians^13^,^14^. However, in this study the knowledge scores of assistant chief doctors and doctors in charge were not significantly increased relative to doctors with junior title, which needed more attention and further research.

The survey instrument used in this study for assessing knowledge of antibiotic use has been widely adopted in other countries^13^,^14^,^15^. Importantly, in this study, the questionnaire was distributed and completed on-site, precluding introduction of bias due to consultancy of peers or reference to published resources. Therefore, we believe that the results of the study reflect the true knowledge of the doctors’ surveyed. However, because our survey was conducted at only one city in China, the results must be generalized with caution.

Some research pointed out that lack of knowledge and training regarding antibiotics may attribute to the inappropriate use of antibiotics^26^,^27^. Recently a large-scale retrospective cohort study showed that physicians’ attitudes and knowledge determine the quality of prescription of antibiotics^28^. Therefore, understanding the doctors’ knowledge on antibiotic use and the associated factors is crucial to make better efforts to promote appropriate antibiotic prescribing. This study found that doctors have suboptimal knowledge about antimicrobials in China. Although ongoing education was partly effective in enhancing doctors’ knowledge on antibiotic use, the effect remains to be further improved. In addition, doctors coming from primary healthcare institutions had poorer knowledge on antibiotic use, which indicated that more targeted interventions and education programs are urgently needed in these hospitals.

## Methods

### Survey instrument

A self-administered questionnaire was used in the present study, which was developed according to a previous questionnaire used in the US, Peru, and Congo^13^,^14^,^15^, and further adapted to the setting in China. Prior to release, the questionnaire was reviewed by a team of five infectious diseases physicians to assess the relevance and wording of the questions as well as accuracy of the translation into Chinese. Then, the questionnaire was pilot-tested on 20 doctors to ensure that the questions were clear and understandable to all participants. The questionnaire was divided into three sections, namely; socio-demographic information, knowledge on antibiotic use and continuing education about antibiotics. Socio-demographic information included gender, age, educational level, specialty and working age. Doctors’ knowledge was assessed by ten antimicrobial questions about the clinical indications, spectrum, and pharmacology of antibiotics. In summary, three questions addressed the choice of antibiotics for treating acute diarrhea, upper respiratory tract infection and sepsis in a patient with impaired renal function; two questions addressed the safety of antibiotics for pregnant women and children; three questions addressed the spectrum of antibiotics and two questions addressed the pharmacology of antibiotics. Continuing education was also investigated by one question that addressed training in antibiotic use. “During the last year, did you receive any training on antibiotic use?”.

### Sampling design and data collection

This is a cross-sectional study. Data were collected from October 2012 to October 2013 in the city of Taiyuan, Shanxi Province (Central China). There were ten tertiary hospitals, thirteen secondary hospitals and sixty-two primary healthcare facilities in Taiyuan in 2012. Tertiary hospitals are the highest level with the best medical equipment and technology, while primary healthcare facilities are the lowest level including community health centers and township hospitals. A stratified cluster random sampling method was employed in the present study. In the first stage, hospitals were divided into three groups according to their hierarchy level, and then two tertiary hospitals, two secondary hospital and twelve primary healthcare facilities were randomly sampled from each hospital group. In the second stage, all licensed medical doctors from general practice, internal medicine, surgery, gynecology and obstetrics, pediatrics and stomatology in the selected hospitals were invited to participate in the survey. Medical doctors from psychiatry, radiology, ophthalmology and anesthesiology were not included as they do not routinely prescribe antibiotics. Considering that doctors have holidays by turns, we went to each selected hospital several times to ensure that all licensed doctors from the above department had a chance to be invited to participate in survey to collect more representative data. Questionnaires were distributed on site during working time by postgraduate students. In order to accurately assess doctors’ knowledge on antibiotic use, participants were asked to respond immediately without referring to the literature or consulting others. Additionally, participants were asked to make a written commitment not to disclose the questions to their colleagues when they signed the written informed consent.

### Statistical analysis

All statistical procedures were performed using the Statistical Analysis System

(SAS) 9.4 for Windows (SAS Institute Inc., Cary, NC, USA). Each antimicrobial question was given a score of one point if the doctor selected the correct answer, and a score of zero if the answer was incorrect. The cumulative score of the ten antimicrobial questions was then calculated to assess doctors’ knowledge on antibiotic use. Descriptive analyses included means for continuous variables and percentages for categorical data. A generalized linear regression model was used to examine the associations of independent variables with doctors’ antibiotic knowledge scores. All comparisons were two-tailed. The significance threshold was a *P* value of <0.05.

This study was performed in accordance with the principles of the Declaration of Helsinki and approved by the Research Ethics Committee of the Tongji Medical College, Huazhong University of Science and Technology, Wuhan, China. Written informed consent was provided for each respondent, and the identifying details of all study subjects were kept confidential.

## Additional Information

**How to cite this article**: Bai, Y. *et al*. Factors associated with doctors' knowledge on antibiotic use in China. *Sci. Rep.* **6**, 23429; doi: 10.1038/srep23429 (2016).

## Figures and Tables

**Table 1:** Descriptive statistics for characteristics and knowledge score on antibiotics of the study participants.

Variables	N	%	Knowledge score (mean ± SD*)
**Total**	761	100	6.29 ± 1.79
**Age (mean ± SD)**	35.55 ± 8.49		–
**Gender**			
Male	288	37.84	6.13 ± 1.89
Female	473	62.16	6.38 ± 1.73
**Education level**			
Associate degree§ or vocational diploma¶	71	9.33	6.61 ± 2.02
Bachelor degree	438	57.56	6.46 ± 1.77
Master degree or higher	252	33.11	5.90 ± 1.70
**Specialty**			
Internal medicine	244	32.06	6.59 ± 1.83
Surgery	156	20.50	6.08 ± 1.79
Gynaecology and obstetrics	93	12.22	6.51 ± 1.51
Pediatrics	64	8.41	5.92 ± 1.26
Stomatology	69	9.07	6.20 ± 1.75
General Practice	135	17.74	6.03 ± 2.05
**Qualification title**			
Chief doctors	67	8.80	6.94 ± 1.42
Assistant chief doctors	107	14.06	6.55 ± 1.53
Doctors in charge	216	28.38	6.13 ± 1.77
Doctors with junior title	371	48.75	6.18 ± 1.90
**Hospital level**			
Tertiary hospital	322	42.31	6.77 ± 1.81
Secondary hospital	174	22.86	6.28 ± 1.89
Primary healthcare facilities	265	34.82	5.71 ± 1.53
**Receive education on antibiotic**			
Yes	691	90.80	6.40 ± 1.75
No	70	9.20	5.14 ± 1.80
**Working duration in hospitals (years, mean** ± **SD)**	10.75 ± 9.45		−

^*^SD = standard deviation.

^§^An associate degree requires 3 years of education in college after graduation from senior middle school (grade year 10 to year 12), or 5 years of education in college after graduation from junior middle school (grade year 7 to year 9).

^¶^A vocational diploma requires 2 years of education in vocational schools after graduation from senior middle school, or 3 years of education in vocational schools after graduation from junior middle school.

**Table 2:** Generalized linear model analysis for the factors associated with physician’s knowledge on antibiotics (N = 761).

Parameter	Estimate	SE	t	*P* value
**Intercept**	7.23	0.73	9.88	<0.01
**Age**	0.03	0.02	1.19	0.24
**Gender**				
Male	0.00	0.00	–	–
Female	0.13	0.15	0.91	0.36
**Education level**				
Associate degree or vocational diploma	0.00	0.00	–	–
Bachelor degree	−0.20	0.22	−0.88	0.38
Master degree or higher	−0.15	0.28	−0.55	0.58
**Specialty**				
Internal medicine	0.00	0.00	–	–
Surgery	−0.34	0.19	−1.81	0.07
Gynaecology and obstetrics	−0.33	0.21	−1.56	0.12
Pediatrics	−0.60	0.25	−2.39	**0.02**
Stomatology	−0.68	0.23	−2.88	**<0.01**
General Practice	−0.70	0.18	−3.85	**<0.01**
**Qualification title**				
Doctors with junior title	0.00	0.00	–	–
Doctors in Charge	−0.18	0.16	−1.14	0.25
Assistant chief doctors	0.28	0.22	1.25	0.21
Chief doctors	0.71	0.25	2.79	**0.01**
**Hospital level**				
Tertiary hospital	0.00	0.00	–	–
Secondary hospital	−0.66	0.17	−3.91	**<0.01**
Primary healthcare facilities	−1.18	0.20	−5.98	**<0.01**
**Receive training on antibiotic use**				
Yes	0.00	0.00	–	–
No	−1.24	0.22	−5.67	**<0.01**
**Working duration in hospitals**	−0.01	0.02	−0.59	0.55
